# Risk Screening of the Non-Native Fish in the Jiulong River Basin of Southeast China

**DOI:** 10.3390/ani15040461

**Published:** 2025-02-07

**Authors:** Shilong Feng, Xindong Pan, Jiaqiao Wang, Wenjuan Liu, Yapeng Hui, Guangzhao Wang, Kai Liu, Jun Li, Haoqi Xu, Lin Lin, Xu Wang, Zhiqiang Wu, Liangmin Huang, Fenfen Ji

**Affiliations:** 1 Fisheries College, Jimei University, Xiamen 361021, China; 202312951064@jmu.edu.cn (S.F.); panxindong@jmu.edu.cn (X.P.); skyofstar1@jmu.edu.cn (J.W.); 202412951041@jmu.edu.cn (Y.H.); 202221063031@jmu.edu.cn (G.W.); liukai1218@jmu.edu.cn (K.L.); lijun1982@jmu.edu.cn (J.L.); xhqcurious@163.com (H.X.); 202411710015@jmu.edu.cn (L.L.); 202412951039@jmu.edu.cn (X.W.); 202321063062@jmu.edu.cn (Z.W.); 2 FuJian Provincial Key Laboratory of Marine Fishery Resources and Eco-Environment, Xiamen 361021, China; 3 Institute of Urban Environment, Chinese Academy of Sciences, Xiamen 361024, China; liuwj1978@163.com

**Keywords:** fish community, diversity conservation, AS-ISK, ecological risk, tilapia

## Abstract

Non-native fish species introduced into new areas, especially when they develop into large populations, pose a threat to native fauna. Understanding the current status of the fish community and invasion risks of non-native fish are essential for invasive species control and diversity conservation. The community structure of fish and a risk assessment on 10 non-native fish species were systematically assessed in the Jiulong River Basin, China, in January, April, and July of 2024. The species richness, with 105 species, showed a notable decrease compared to the 124 species recorded in 1975, while the number of non-native species has increased from zero to ten. Furthermore, the non-native fish species, *Coptodon zillii* and *Sarotherodon galilaeus*, have become dominant species, with IRI values of 4038.43 and 1180.30, respectively. The AS-ISK established risk thresholds for BRA and BRA + CCA as 29.5 and 35.5, respectively, identifying 70% of the non-native fish species as high-risk species, including *C. zillii*, *S. galilaeus*, *Oreochromis niloticus*, *Clarias batrachus*, *Hypostomus plecostomus*, and *Oreochromis aureus*. This study indicates that the fish species richness in the Jiulong River Basin has declined, with *C. zillii* and *S. galilaeus* becoming dominant and posing high ecological risks to the native fish community. In addition, targeted fishing during the breeding season should be used to control the population of tilapia and restore fish diversity.

## Simple Summary

Freshwater habitats, despite covering less than 1% of the Earth’s surface, harbor over 40% of the global fish diversity. However, the invasion of non-native fish species has significantly reduced biodiversity and disrupted ecosystems worldwide. The Jiulong River Basin, the second-largest river in the Fujian Province and home to over 12 million people, has witnessed a decline in fish diversity from 124 species in 1975 to 105 species in 2024, alongside an increase in non-native species from zero to ten. Using the Aquatic Species Invasiveness Screening Kit (AS-ISK), this study evaluated the invasion risks of non-native fish and revealed that 70% of the invasive species in the basin are high-risk, posing severe threats to native fish communities. Tilapia species, which dominate the basin, were identified as high-risk invasive species requiring urgent management. This study systematically assessed fish communities and non-native species, established a prioritized control framework, and proposed actionable recommendations for invasive fish management. The findings provide references for methods and strategies to control invasive species in other freshwater ecosystems, contributing to biodiversity conservation and ecological balance.

## 1. Introduction

Fish are an indispensable component of ecosystem functions and biodiversity, playing a key role in maintaining water environment health, ecosystem balance, and serving as ecological indicators [1]. Despite covering less than 1% of the Earth’s surface, freshwater habitats harbor over 40% of the global fish diversity [2]. However, in recent years, the invasion of non-native fish species has caused a significant decline in fish diversity in freshwater ecosystems around the world [3]. Approximately 10% to 15% of freshwater ecosystems worldwide are affected and troubled by non-native fish species [4]. China has the largest number of non-native fish in the world, totaling 439 species, among which 53 species have established self-sustaining populations in the freshwater ecosystems [5]. These introductions have led to a 7.0% increase in the homogenization of the national fish community, making it one of the primary causes of biodiversity loss [6]. Therefore, the management of non-native fish species is a pressing global issue, and the control order of non-native species, along with targeted strategies, is crucial for the effective management of non-native fish species in the freshwater ecosystems.

Generally, natural water bodies are likely to be affected by the presence of two or more non-native species [7,8]. Risk assessments of non-native species serves as the primary foundation for determining the control order of non-native species [9,10]. The initial risks assessments of non-native fish primarily relied on models developed by researchers. For instance, Kolar and Lodge developed a quantitative model based on the biological traits of invasive fish to predict potential fish invaders in the Great Lakes of North America [11]. Singh et al. developed a model based on the catch numbers and trophic status of fish to assess the risk of non-native fish in India’s Ganges River, demonstrating that the fish invasions are a significant driver of biodiversity loss [12]. Additionally, Simonovi et al. used the Fish Invasiveness Screening Kit (FISK) to screen 43 non-native fish species, identifying 3 low-risk species, 10 medium-risk species, and 30 high-risk species [13]. In recent years, with the development of technology in methodology, the use of the Aquatic Species Invasiveness Screening Kit (AS-ISK) method has been increasingly applied and developed [14]. The AS-ISK is the next-generation decision support tool of FISK [15,16]. Interesova et al. applied the AS-ISK to screen 31 existing and potential invasive fish species, identifying 12 invasive species and assessing their risks. This provided significant support for invasive fish policies and management in the West Siberian Plain and the Ob River basin [9]. Ge et al. used AS-ISK to determine the invasion risks of non-native fish in the Yunnan Province, China, assessing 21 species as high risk, 13 as medium risk, and 3 as low risk, thereby reliably evaluating the invasion risks and establishing a priority order for non-native fish controls in the Yunnan Province [10]. In general, AS-ISK can accurately distinguish the risk level of non-native species and provide the priority level of non-native species management for policy and decision-makers.

Invasive fish species pose a significant global threat to aquatic ecosystems, resulting in biodiversity loss, habitat degradation, and ecological imbalances [17]. Effective management strategies for addressing these issues generally fall into three categories: physical, chemical, and biological methods. For example, physical removal through targeted fishing has been employed in various regions to reduce invasive populations [18]. Chemical approaches, such as the use of piscicides, have also been applied, though with potential non-target effects [19]. Biological control, involving species like mosquitofish (*Gambusia* spp.), has shown mixed outcomes, as these interventions may sometimes lead to unintended ecological consequences [20]. All effective restoration and management efforts, however, must be grounded in comprehensive assessments of fish diversity and invasion risks. Risk assessment tools, such as the Aquatic Species Invasiveness Screening Kit (AS-ISK) [14,15], offer valuable frameworks for evaluating invasion risks. These tools provide a systematic approach to identifying high-risk species, prioritizing management efforts, and allocating resources effectively.

The Jiulong River Basin is the second largest river in the Fujian Province and serves as a vital water source for 12 million people in Longyan, Zhangzhou, and Xiamen city. The basin covers five cities with a drainage area of 1.48 km^2^, and its geographical position lies between 116.78° E–118.03° E and 24.22° N–25.85° N [21]. The fish community in the Huaan of the Jiulong River basin was surveyed in 2013; however, a comprehensive survey of the entire basin and a systematic risk assessment of the non-native fish was not conducted [22]. The current status of fish community and invasion risks of non-native fish were assessed in January (the low flow season), April (the normal flow season) and July (the high flow season) 2024 in the Jiulong River Basin. The objectives of this study are as follows: (1) to systematically survey the fish community and the current status of non-native fish species in the Jiulong River Basin; (2) to evaluate the risks of non-native fish species using AS-ISK and provide recommendations for non-native fish control; and (3) to provide references of methods and strategies for the control of non-native species in other freshwater ecosystems.

## 2. Materials and Methods

### 2.1. Study Area

**Figure 1:**
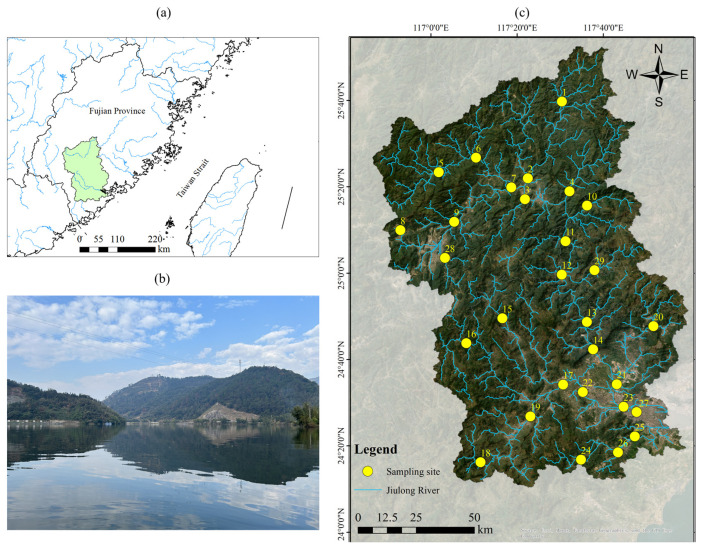
Map of the study (risk screening) area (Jiulong River Basin, China). (**a**) The geographical location of the Jiulong River Basin; (**b**) a real-world image of the upstream region of the Jiulong River Basin; (**c**) distribution map of the sampling sites in the Jiulong River Basin.

The Jiulong River basin is a subtropical coastal watershed in southeast China with a drainage area of 14,741 km^2^ (Figure 1). The mean annual runoff is 1010 mm and the annual precipitation ranges from 1400 mm to 1800 mm and nearly 75% of the precipitation occurs from April to September. The mean annual temperature is 19.9–21.1 °C. Two major tributaries, the North River and West River, cover nearly 95% of the total area of the Jiulong River basin [23]. The Jiulong River Basin has significant ecological service functions, yet the fish communities and invasion status of non-native fish have not been systematically assessed. In this study, the current status of the fish community and the risk assessment of the non-native fish was investigated at 29 sites in the Jiulong River Basin in January (the low flow season), April (the normal flow season) and July (the high flow season) 2024.

### 2.2. Sample Collection and Appraisal

Samples for the fish were obtained following the Technical guidelines for biodiversity monitoring—inland water fish (HJ 710.7—2014) [24]. Fish samplings were conducted in the pelagic area by gill netting, and in the benthic area by traps. Three gillnets of each size and three traps were set at each site [25]. The gillnets (35.0 m length × height 1.2 m; 50.0 m length × 1.5 m height; 65.0 m length × height 1.2 m, with uniform stretched mesh sizes of 30.0, 60.0, and 120.0 mm) were set before dusk and lifted after dawn to assure the fishing of the likely maximum activity periods for all of the fish species at each site. To ensure more comprehensive catches, we deployed the gill nets at varying depths across the water column to target fish in the surface, mid-water, and bottom layers. The traps, measuring 50.0 m in length and 30 cm in diameter, were constructed from durable nylon netting with a mesh size of 15 mm. They were set for 24 h at each site to ensure a sufficient time for capturing representative fish species.

Fish specimens were collected, observations recorded, biological data measured (fish quantity and weight) and the surviving fish were released back into the capture area [26]. Sample identification was conducted using the reference book *Fujian Fish* [27], which provides comprehensive taxonomic keys, morphological descriptions, and illustrations for fish species commonly found in the Fujian Province of China. This resource is widely recognized as an authoritative guide for ichthyological studies in the region.

### 2.3. Data Analysis

#### 2.3.1. Analysis of Dominant Species

Dominant species were assessed using the Pinkas Index of relative importance (*IRI*) [28].

IRI=(N+W) F

where *N* is the numerical percentage of each category in the catch, *W* is the weight percentage of each category in the catch, and *F* is the ratio of the number of occurrences of a category in the sample to the number of surveys. Species with an IRI > 1000 were regarded as dominant species, those with 100 < IRI < 1000 as important species, those with 10 < IRI < 100 as common species, those with 1 < IRI < 10 as occasional species, and those with IRI < 1 as rare species.

#### 2.3.2. Risk Screening Analysis

The AS-ISK (The free download link for AS-ISK is available at www.cefas.co.uk/nns/tools/ accessed on 24 December 2024.) consists of 49 basic questions that examine the biogeographical and biological aspects of the taxon being screened, resulting in a Basic Risk Assessment (BRA) score. The AS-ISK includes six additional Climate Change Assessment (CCA) questions that requires the assessor to evaluate how future climatic conditions may influence the Basic Risk Assessment (BRA) score. These questions focus on assessing the risks related to the introduction, establishment, dispersal, and potential impact of non-native species under changing climatic scenarios, resulting in a combined BRA + CCA score [29]. To ensure a valid AS-ISK assessment, assessors must provide three components for each question: (1) a response, (2) a justification for the response, and (3) a confidence rank for the response. The corresponding answers are detailed in Supplementary Material S1. These elements collectively ensure that the responses are well supported, leveraging bibliographic sources wherever feasible, and reflect the assessor’s level of certainty based on the available knowledge. The Basic Risk Assessment (BRA) score ranges from −20 to 68, while the combined BRA + CCA score spans from −32 to 80. Confidence levels are ranked on a four-point scale: 1 (low), 2 (moderate), 3 (high), and 4 (very high), providing a measure of reliability for each assessment [14].

The predictive ability of AS-ISK to identify high-risk invasive fish species was assessed using receiver operating characteristic curve analysis (ROC) [30] after calculating BRA and BRA + CCA scores. If sufficient evidence of invasive fish is present, the species may be classified a priori as invasive; if no evidence is found, the species is classified a priori as non-invasive. The ROC curve is a plot of sensitivity versus ‘1-specificity’ (or sensitivity versus specificity) for each threshold, where sensitivity and specificity refer to the proportions of a priori invasive and non-invasive species correctly identified by AS-ISK, respectively. The area under the curve (AUC), a measure of the accuracy of calibration analyses, typically ranges from 0.5 to 1.0—the closer it is to 1.0, the better the ability to distinguish between invasive and non-invasive species [31]. If the AUC is equal to 1.0, then the test is 100% accurate, because both sensitivity and specificity are 1.0, and there are neither ‘false positives’ (a priori non-invasive species classified as high risk, hence invasive) nor ‘false negatives’ (a priori invasive species classified as low risk, hence non-invasive). Conversely, if the AUC is equal to 0.5, then the test is 0% accurate as it cannot discriminate between ‘true positives’ (a priori invasive species classified as high risk, hence invasive) and ‘true negatives’ (a priori non-invasive species classified as low risk, hence non-invasive) [9]. Following ROC curve analysis, the optimal AS-ISK threshold that maximizes true positives and minimizes false positives was determined using Youden’s index and the ‘default’ threshold was set to 1 to differentiate between low and medium risk species [32]. ROC curve analyses were performed using SPSS 26.0, and specificity confidence intervals were calculated using 2000 bootstrap replicates along the entire range of sensitivity points (i.e., 0 to 1 with an interval of 0.1) [33]. Threshold intervals are indicated by statistically appropriate brackets (‘]’ and ‘[’). Based on the confidence level (CL) assigned to each response (see Risk Screening), a confidence factor (CF) can be derived. A confidence factor (CF) is obtained as follows:

$$CF=\Sigma (CL_{Q_{i}})/(4\times 55)(i=1,\ldots ,55)$$

where CL_Qi_ is the confidence level for the ith Question (Qi), 4 is the maximum achievable value for confidence (i.e., very high, see above) and 55 is the total number of questions. Based on the 49 Qs comprising the BRA and the six Qs comprising the CCA, the CL_BRA_ and CL_CCA_ are also computed (out of the CL_Total_ for all 55 Qs) [34].

#### 2.3.3. Statistical Analyses

Statistical analyses were conducted using SPSS 26.0 software and Excel 2019. The pie charts of species composition, bar charts representing the invasion level of non-native fish species, and line charts indicating confidence levels, were generated in Origin 2022. A one-way ANOVA was used to analyze the differences in fish abundance across different periods. The ROC curve plot was generated in GraphPad Prism 8, and its statistical significance was evaluated using the area under the curve (AUC) and a significance threshold of *p* < 0.05.

## 3. Results

### 3.1. Fish Community Structure in the Jiulong River Basin

**Figure 2:**
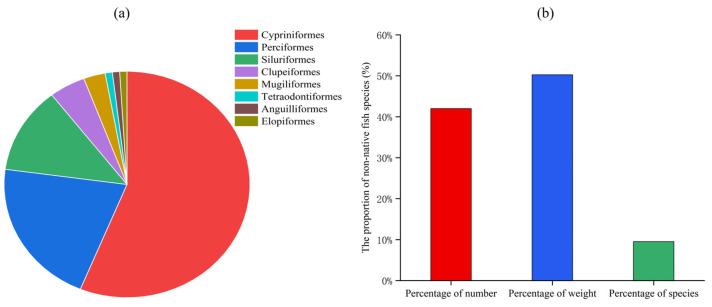
The percentage of order richness in the Jiulong River Basin (**a**) and the individual number, weight, and species richness percentage of non-native fish in the Jiulong River Basin (**b**).

In 2024, a total of 105 fish species were identified in the Jiulong River Basin across the low flow season, normal flow season, and high flow season (Supplementary Material S2, Table S1). Among these, 59 species belonged to the Cypriniformes (56.19%), 22 species to the Perciformes (20.95%), 13 species to the Siluriformes (12.38%), 5 species to the Clupeiformes (4.76%), 3 species to the Mugiliformes (2.86%), and 1 species each to the Tetraodontiformes, Elopiformes, and Anguilliformes (0.95% each) (Figure 2a). In the Jiulong River Basin, a total of 10 non-native species were identified, accounting for 9.52% of the total species and belonging to three orders, five families, and six genera (Table 1). These species were *Coptodon zillii*, *Sarotherodon galilaeus*, *Oreochromis niloticus*, *Oreochromis aureus*, *Hypostomus plecostomus*, *Clarias batrachus*, *Parachromis managuensis*, *Oreochromis mossambicus*, *Cirrhinus mrigala*, and *Macropterus salmoides*. The number of non-native species surveyed in the Jiulong River Basin accounted for 42.00% of the total, and their weight represented 50.26% of the total (Figure 2b). The total number of individuals caught, along with the species composition, is provided in Supplementary Material S2, Table S2, which details the abundance of each fish species recorded during the sampling period.

A total of 64 species were recorded during the low flow season, 81 species during the normal flow season, and 63 species during the high flow season (Supplementary Material S2, Table S1). Furthermore, Cypriniformes and Perciformes are the two most species-rich orders, accounting for 56.25% and 17.19% of the species in the low flow season, 55.56% and 19.75% in the normal flow season, and 58.73% and 22.22% in the high flow season, respectively (Supplementary Material S2, Figures S1–S3). Additionally, eight, eight, and nine non-native species were found in the low flow season, normal flow season, and high flow season, respectively (Table 1). The number of non-naive species accounted for 37.91%, 38.79%, and 51.53% during the low flow season, normal flow season, and high flow season, respectively (Supplementary Material S2, Figures S4–S6). The weight of non-native species accounts for 42.77%, 53.72%, and 52.15% of the total weight during the low flow season, normal flow season, and high flow season, respectively (Supplementary Material S2, Figures S4–S6). A one-way ANOVA showed no significant differences in the species richness per site among the three hydrological periods (F = 3.928, df = 2, *p* > 0.05).

### 3.2. The Abundance Distribution of Non-Native Fish Species

**Figure 3:**
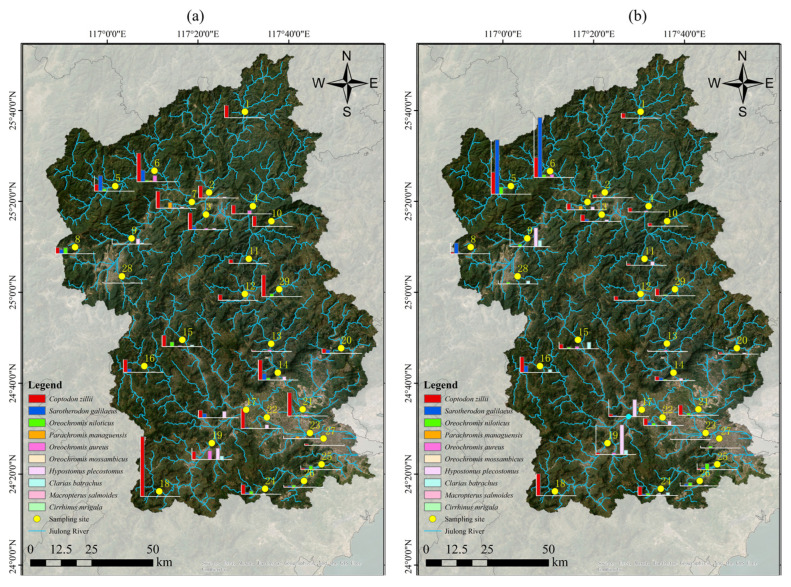
(**a**) Abundance distribution of non-native fish species by number in the Jiulong River Basin; (**b**) Abundance distribution of non-native fish species by weight in the Jiulong River Basin.

In this study, 29 sites in the Jiulong River Basin were surveyed during the low, normal, and high flow seasons of 2024. A total of 2072 invasive fish were captured, weighing 188,609.85 g. Among them, *C. zillii* accounted for 1311 individuals, weighing 62,275.01 g; *S. galilaeus* for 248 individuals, 57,421.23 g; *O. niloticus* for 174 individuals, 17,592.35 g; *P. managuensis* for 45 individuals, 2399.1 g; *O. aureus* for 101 individuals, 4629.29 g; *O. mossambicus* for 2 individuals, 23.15 g; *H. plecostomus* for 152 individuals, 33,539.22 g; *C. batrachus* for 36 individuals, 10,410.7 g; *M. salmoides* for 2 individuals, 68.8 g; and *C. mrigala* for 1 individual, 84.5 g. Additionally, *C. zillii* was found at all 29 sites in the Jiulong River Basin, while *S. galilaeus*, *O. niloticus*, *P. managuensis*, *O. aureus*, *H. Plecostomus* and *C. batrachus* were densely distributed throughout the basin (Figure 3).

### 3.3. Relative Importance Value of Non-Native Species

Among the ten non-native species, *C. zillii* and *S. galilaeus* were identified as dominant species with the IRI value of 4038.43 and 1180.30, respectively (Table 2). *O. niloticus*, *H. plecostomus*, and *O. aureus* were classified as important species with the IRI value of 688.98, 620.28 and 116.42, respectively. *C. batrachus* and *P. managuensis* were identified as common species with the IRI value of 79.12 and 41.18, respectively. (Table 2). *C. mrigala*, *O. mossambicus*, and *M. salmoides* were identified as rare species with the IRI value of 0.14, 0.30 and 0.38, respectively (Table 2).

Regarding IRI value of non-native species for the three period, *C. zillii* was the dominant species, with an IRI value of 2950.68 in the low flow season. *C. zillii* and *S. galilaeus* were dominant species, with IRI values of 3368.28 and 1178.43, respectively in the normal flow season. *C. zillii* maintained its dominance, with an IRI value of 4552.73 in the high flow season (Table 2).

### 3.4. Risk Assessment of Non-Native Fish

**Figure 4:**
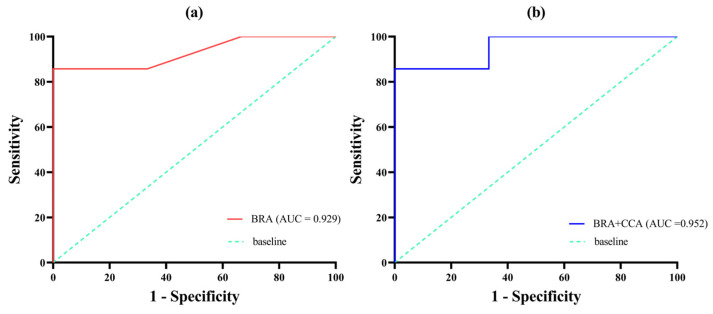
Receiver operating characteristic (ROC) curve for the assessment of 10 non-native fish in the Jiulong River Basin using AS-ISK (BRA score ROC curve (**a**) and BRA + CCA score ROC curve (**b**)).

**Figure 5:**
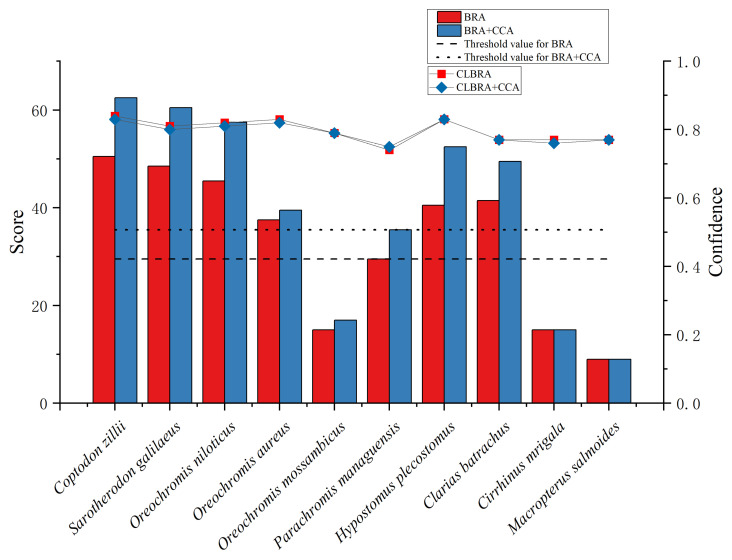
AS-ISK scores (bar graphs) and confidence levels of AS-ISK scores (line graphs) for 10 non-native fish in the Jiulong River Basin. The line across the bars corresponds to the threshold resulting from Youden’s index.

The ROC curve analysis for the BRA score resulted in an AUC of 0.929, while the ROC curve for the combined BRA + CCA score yielded an AUC of 0.952 (Figure 4). Youden’s J provided a threshold of 29.5 for the BRA score and 35.5 for the BRA + CCA score, which were used to calibrate the results of the risk assessment. The calibrated BRA score, respectively, defined low, medium, and high-risk intervals as [−20, 1], [1, 29.5], and [29.5, 68], respectively. For the BRA + CCA score, the low, medium, and high-risk thresholds were [−32, 1], [1.0, 35.5], and [35.5, 80], respectively. Among the ten fish species, 70% (seven species) were classified as high-risk, and 30% (three species) as medium risk, based on both BRA and BRA + CCA threshold results (Table 3; Figure 5). Within the BRA score (≥29.5), the highest-ranked species, from highest to lowest scores, were *C. zillii*, *S. galilaeus*, *O. niloticus*, *C. batrachus*, *H. plecostomus*, and *O. aureus*. In the BRA + CCA score (≥35.5), the order remained the same for these high-scoring species (Table 3).

Across all 55 questions, the average confidence factor for the BRA assessment score was 0.79 ± 0.03, and for the BRA + CCA assessment score, it was 0.79 ± 0.07. The average confidence factor for the assessment scores was greater than 0.75, indicating a moderate to high confidence in all cases.

## 4. Discussion

In this study, the fish communities and risks assessment of non-native fish were estimated in the Jiulong River Basin. Our findings suggested that the fish richness in the Jiulong River Basin has declined with non-native tilapia emerging as the dominant species. Furthermore, 70% of the non-native fish species were identified as high-risk species, which pose high ecological risks to the native fish community. Fish diversity conservation and non-native species control should be prioritized and effectively implemented in the Jiulong River Basin.

### 4.1. The Changes in the Fish Community of the Jiulong River Basin

In this study, we found a total of 105 species (95 native and 10 non-native), belonging to 8 orders, 20 families, and 73 genera, with Cypriniformes as the dominant order, accounting for 56.19%. Lian et al. indicated 124 fish species (with no non-native species detected) were found in the Jiulong River Basin from 1975 to 1979, predominantly Cypriniformes, which accounted for 51.60% [35]. Compared to historical data, the community composition of fish has remained consistent, with Cypriniformes as the main group. The species richness has decreased, and more notably, the species richness of non-native fish has risen from 0 to 10. Similarly, Fang conducted a fish community survey in the Huaan section of the Jiulong River Basin, identifying 20 species, of which three were non-native fish [21]. Both this study and previous research indicate that while the composition of the fish community in the Jiulong River Basin were similar, there has been a decline in species richness and an increase in the number of non-native species. Furthermore, the individual number of non-native species accounted for 42.00% of the total fish population, and their weight made up 50.26% of the total catch. The IRI results further indicated that the non-native species *C. zillii* and *S. galilaeus* are dominant, with values of 4038.43 and 1180.30, respectively. The non-native *O. niloticus*, *H. plecostomus*, and *O. aureus* are identified as important species. Fang also showed that the non-native fish have established populations, in which the non-native fish accounted for 35.96% of the catch by quantity and 30.63% by weight in the Huaan section of the Jiulong River Basin [21]. In summary, this study revealed a decline in the fish species richness and a high degree of fish invasions in the Jiulong River Basin, highlighting the need for a focused attention and management of the invasive species risk control.

### 4.2. Invasion Risk Assessment of Fish in the Jiulong River Basin

The AS-ISK tool has been widely used to assess the invasion risk of non-native fish in multiple regions worldwide [33]. In this study, the AS-ISK tool classified all 10 non-native fish species as medium- to high-risk invasive species. The ROC curve analysis of the BRA score yielded an AUC of 0.929, while the BRA + CCA score resulted in an AUC of 0.952. These values indicate a high confidence level in the AS-ISK tool’s assessment scores, demonstrating its effectiveness for screening the invasion risk of non-native fish species in the Jiulong River Basin. Additionally, the risk thresholds for the Jiulong River Basin, based on the BRA and BRA + CCA scores for the 10 fish species (29.5 and 35.5, respectively), are significantly higher than the global standard risk thresholds proposed by Vilizzi et al., in which the value was 14.7 for BRA and 17.7 for BRA + CCA [33]. There are two possible reasons for the higher BRA and BRA + CCA scores in this study: firstly, the two dominant fish species in the Jiulong River Basin are both tilapia, which possess strong ecological adaptability, including a tolerance to high temperatures, salinity, and pollution [36]. Secondly, over the past 48 years (1971–2018), summer and autumn temperatures and precipitation in Fujian, China, have risen significantly [37], providing more favorable conditions for the survival of non-native fish species, especially tilapia, in the Jiulong River Basin. Similarly, Interesova et al. used AS-ISK to assess the invasion risk in the Ob River Basin on the West Siberian Plain, Russia, where the BRA and BRA + CCA thresholds were 27.5 and 34.75, respectively [9]. Their study also indicated that rising temperatures and the increased tolerance of fish species led to higher risk assessment scores and elevated threshold values. Therefore, both this study and previous research indicate that AS-ISK is suitable for assessing the invasion risk of fish species in the Jiulong River Basin, with risk assessment scores demonstrating accuracy and reliability.

Among the 10 non-native fish species assessed in this study, seven species have been identified as high-risk species. Among these species, *O. niloticus*, *C. gariepinus*, *M. salmoides*, and *C. mrigala* are widely recognized for their economic value due to their extensive use in aquaculture and the fish trade [1,7]. However, despite their economic benefits, the introduction of these species into non-native environments poses significant ecological risks. In the wild, their presence can disrupt local ecosystems by outcompeting native species, altering food webs, and potentially introducing diseases [10]. Tilapia, classified as a dominant species and a high-risk invasive species in the Jiulong River Basin, posed a considerable ecological threat and was ranked as the world’s third most significant invasive species contributing to adverse ecological outcomes [38,39]. Once tilapia becomes the dominant species in an ecosystem, it competes with native species for nutrients and habitat, occupying the nutritional and spatial niches of other fish, thereby affecting the growth and reproduction of native fish species [40,41]. Meanwhile, tilapia has a broad diet, preying on fish eggs, shrimp, crabs, and juvenile fish. Consequently, the overpopulation of tilapia can significantly reduce the biomass of native fish species, threatening fish diversity [42,43]. Additionally, the excretion and nesting activities from tilapia could increase the eutrophication level of the water, deteriorating water quality [44,45]. Gu et al. demonstrated that the invasive *O. niloticus* not only affects the CPUE (catch-per-unit-per-effort) of the native fish community but also inhibited the growth of mud carp with similar prey groups in the main rivers of Guangdong Province, China [46]. Andreu-Soler and Ruiz-Campos found that competitive effects from *C. zillii* over habitat and breeding grounds led to a decline in the population of *Fundulus lima* and a reduction in overall fish diversity [47]. Additionally, both *C. batrachus* and *H. plecostomus* have also been assessed as high-risk invasive species in the Jiulong River Basin, exhibiting similar issues such as preying on fish fry and eggs, and deteriorating water quality [48,49]. However, their current populations and dominance are significantly lower compared to tilapia. Champneys et al. demonstrated that controlling the dominant and high-risk non-native fish is a crucial step in restoring the advantageous position of native fish within the current aquatic system [50]. Thus, our study indicated that 70% of the non-native fish species in the Jiulong River Basin are high-risk, with tilapia being the predominant species. Controlling tilapia populations is crucial for the sustainable development of fish diversity in the Jiulong River Basin.

### 4.3. Recommendations for Non-Native Fish Management

Tilapia, originally native to Africa, is a highly invasive fish species that has spread widely and been introduced to various regions around the world, including Asia, North America, Latin America, and the Middle East etc. [44]. Tilapia was first introduced to China in 1957 for aquaculture purposes [51]. The tilapia has established dominant populations in southern China, including in provinces such as Fujian, Guangdong, Guangxi, Hainan, Guizhou, and Zhejiang etc., having significantly impacting and reducing biodiversity [8]. Therefore, it is imperative to develop effective management strategies to address the overpopulation of tilapia in waters affected by the non-native tilapia. In North America, the United States has legalized the unrestricted fishing of non-native fish to control invasive species [52]. In Australia, methods such as nest destruction, male control techniques, and electrofishing have been used to reduce the number of invasive fish [53]. However, these methods are costly and have proven insufficient for the complete eradication of tilapia. Ma et al. used a specific concentration of the chemical “Mie fei ling” to eliminate *O. niloticus* in small ponds of China, achieving significant results, though the residual toxicity and long-term impact on natural water bodies remain unclear [19]. Given the current decline in species richness, coupled with the abundance of tilapia and its high ecological risk in the Jiulong River Basin, targeted fishing is recommended. Regular harvesting of tilapia fry, juveniles, and adults can effectively reduce the numbers of tilapia, improve habitat and nutritional niches for other fish species, and enhance the stability of the food web [54]. Thus, we suggest that targeted fishing during the breeding season should be used to control the population of tilapia and restore fish diversity for the Jiulong River Basin.

## 5. Conclusions

This study provided a comprehensive assessment of fish community and non-native fish, established a prioritized control order for non-native species, and offered recommendations for the effective management of non-native fish. The results indicated that, compared to historical data, the fish species richness has declined, and ten new non-native species have been introduced. Furthermore, the non-native fish species, *C. zillii* and *S. galilaeus*, have become dominant species. In addition, 70% of the ten invasive species are high-risk, posing high ecological risks to the native fish community. Controlling the further spread of the non-natitive population, especially for tilapia, is crucial for the sustainable development of fish diversity in the Jiulong River Basin. Targeted fishing during the breeding season is suggested to control the population of tilapia, and restore fish diversity. This study provides reference for the method of risk assessment and the management of non-native species, and particularly offers recommendations for effective control measures against tilapia.

## Figures and Tables

**Table 1:** The appearance of non-native fish species in different flow seasons of the Jiulong River Basin.

Species	Total	The Low Flow Season	The Normal Flow Season	The High Flow Season	Native Region
*Coptodon zillii*	+	+	+	+	Africa
*Sarotherodon galilaeus*	+	+	+	+	Africa
*Oreochromis niloticus*	+	+	+	+	Africa
*Hypostomus plecostomus*	+	+	+	+	Africa
*Oreochromis aureus*	+	+	+	+	Africa
*Clarias batrachus*	+	+	+	+	Central America
*Parachromis managuensis*	+	+	+		North America
*Macropterus salmoides*	+	−	−	+	South America
*Oreochromis mossambicus*	+	+	−	+	Southeast Asia
*Cirrhinus mrigala*	+	−	+	−	India

“+”: The presence of non-native fish during the flow seasons. “−”: Non-native fish not found during this flow seasons.

**Table 2:** The IRI value of Non-native fish in the Jiulong River Basin.

Species	Total	The Low Flow Season	The Normal Flow Season	The High Flow Season
*Coptodon zillii*	4038.43	2950.68	3368.28	4552.73
*Sarotherodon galilaeus*	1180.30	806.49	1178.43	481.23
*Oreochromis niloticus*	688.98	405.59	261.69	711.77
*Carassius auratus*	641.68	788.34	354.33	266.84
*Hypostomus plecostomus*	620.28	176.98	442.30	676.05
*Distoechodon compressus*	618.86	447.44	404.15	409.24
*Hemiculter leucisculus*	566.08	186.15	518.18	350.24
*Hypophthalmichthys molitrix*	343.77	191.72	62.53	503.97
*Pseudogobio vaillantii*	261.71	446.10	119.27	10.49
*Pelteobagrus fulvidraco*	252.38	17.93	203.96	197.50
*Acrossocheilus wenchowensis*	201.50	179.35	105.49	134.48
*Rhinogobius giurinus*	171.65	19.54	195.05	52.23
*Oreochromis aureus*	116.42	36.75	8.12	179.84
*Clarias batrachus*	79.12	22.18	47.30	87.88
*Parachromis managuensis*	41.18	6.90	29.89	16.74
*Macropterus salmoides*	0.38	-	-	1.54
*Oreochromis mossambicus*	0.30	0.29	-	0.31
*Cirrhinus mrigala*	0.14	-	0.32	-
*Others*	781.97	536.60	446.67	508.17

Total: The overall IRI value across the three distinct flow seasons. “-”: Non-native fish not found during this flow seasons.

**Table 3:** Non-native fish assessed with the Aquatic Species Invasiveness Screening Kit (AS-ISK) for the Julong River Basin.

Species	A Priori Categorization	Results			Confidence			
		BRA	Level	BRA + CCA	Level	BRA + CCA	BRA	CCA
*Coptodon zillii*	Y	50.5	High	62.5	High	0.83	0.84	0.75
*Sarotherodon galilaeus*	Y	48.5	High	60.5	High	0.80	0.81	0.75
*Oreochromis niloticus*	Y	45.5	High	57.5	High	0.81	0.82	0.75
*Oreochromis aureus*	Y	37.5	High	39.5	High	0.82	0.83	0.75
*Oreochromis mossambicus*	N	15	Medium	17	Medium	0.79	0.79	0.75
*Parachromis managuensis*	Y	29.5	High	35.5	High	0.75	0.74	0.75
*Hypostomus plecostomus*	Y	40.5	High	52.5	High	0.83	0.83	0.83
*Clarias batrachus*	Y	41.5	High	49.5	High	0.77	0.77	0.75
*Cirrhinus mrigala*	N	15	Medium	15	Medium	0.76	0.77	0.75
*Macropterus salmoides*	N	9	Medium	9	Medium	0.77	0.77	0.75

A priori categorization (N: non-invasive; Y: invasive); results (BRA: Basic Risk Assessment, BRA + CCA: BRA + Climate Change Assessment, Delta: BRA + CCA score minus BRA score); Confidence (CL: confidence level); Outcome (Low: score < 1, Medium: 1 ≤ score < 29.5, High: score ≥ 35.5).

## Data Availability

The original contributions presented in this study are included in the article/Supplementary Material. Further inquiries can be directed to the corresponding author(s).

## Supplementary Materials

The following supporting information can be downloaded at https://www.mdpi.com/article/10.3390/ani15040461/s1. Supplementary Material S1: This file includes ten sheet. The titles of the sheets are listed as following: Screening 1. Invasion risk assessment report for *Clarias batrachus*. Screening 2. Invasion risk assessment report for *Hypostomus plecostomus*. Screening 3. Invasion risk assessment report for *Parachromis managuensis.* Screening 4. Invasion risk assessment report for *Cirrhinus mrigala*. Screening 5. Invasion risk assessment report for *Oreochromis mossambicus*. Screening 6. Invasion risk assessment report for *Oreochromis aureus*. Screening 7. Invasion risk assessment report for *Oreochromis niloticus*. Screening 8. Invasion risk assessment report for *Coptodon zillii*. Screening 9. Invasion risk assessment report for *Macropterus salmoides*. Screening 10. Invasion risk assessment report for *Sarotherodon galilaeus*. Supplementary Material S2: This file includes six supplementary figures and two table. The table and figure titles are as follows: Table S1. List of fishes in the Jiulong River Basin. Table S2. Fish Composition of the Jiulong River Basin. Figure S1. The percentage of order richness during the low flow season in the Jiulong River Basin. Figure S2. The percentage of order richness during the normal flow season in the Jiulong River Basin. Figure S3. The percentage of order richness during the high flow season in the Jiulong River Basin. Figure S4. The individual number, weight and species richness Percentage of non-native fish during the low flow season in the Jiulong River Basin. Figure S5. The individual number, weight and species richness Percentage of non-native fish during the normal flow season in the Jiulong River Basin. Figure S6. The individual number, weight and species richness Percentage of non-native fish during the high flow season in the Jiulong River Basin.
