# Supplementary material for: Risk Screening of the Non-Native Fish in the Jiulong River Basin of Southeast China
Source: Animals (Basel). 2025 Feb 7;15(4):461. doi: 10.3390/ani15040461 (PMC11851879; doi:10.3390/ani15040461)
Supplement: Supplementary file 1 [file animals-15-00461-s001.zip › Supplementary material S2.pdf]

Article

Risk Screening of the Non-Native Fish in the Jiulong River Basin of Southeast China

Shilong Feng [1,2,†], Xindong Pan [1,2,†], Jiaqiao Wang [1,2], Wenjuan Liu [3], Yapeng Hui [1,2], Guangzhao Wang [1,2], Kai Liu [1,2], Jun Li [1,2], Haoqi Xu [1,2], Lin Lin [1,2], Xu Wang [1,2], Zhiqiang Wu [1,2], Liangmin Huang [1,2,*] and Fenfen Ji [1,2,*]

[1] Fisheries College, Jimei University, Xiamen 361021, China
[2] FuJian Provincial Key Laboratory of Marine Fishery Resources and Eco-Environment, Xiamen 361021, China
[3] Institute of Urban Environment, Chinese Academy of Sciences, Xiamen 361024, China
***** Correspondence: lmhuang@jmu.edu.cn (L.H.); 202261000140@jmu.edu.cn (F.J.)
† These authors contributed equally to this work.

Table S1. List of fishes in the Jiulong River Basin.

Species	Low flow season	Normal flow season	High flow season
Ⅰ **Cypriniformes**			
1. *Cyprinus carpio*	+	+	+
2. *Sarcocheilichthys sinensis fukiensis*		+	
3. *Sarcocheilichthys sinensis*	+		
4. *Sarcocheilichthys kiangsiensis*	+		+
5. *Sarcocheilichthys nigripinnis*		+	+
6.*Carassius auratus*	+	+	+
7.*Abbottina rivularis*	+		
8.*Pseudobrama simoni*		+	+
9.*Onychostoma barbatulum*		+	
10.*Acrossocheilus fasciatus*			+
11. *Acrossocheilus kreyenbergii*		+	
12. *Acrossocheilus wenchowensis*	+	+	+
13. *Acrossocheilus fasciatus*		+	+
14. *Acrossocheilus hemispinus*	+		
15. *Rhodeus sinensis*		+	+
16. *Aphyocypris chinensis*		+	
17.*Mylopharyngodon piceus*			+
18. *Ctenopharyngodon idellus*	+	+	+
19. *Squaliobarbus curriculus*		+	+
20. *Hemiculter leucisculus*	+	+	+
21. *Hemiculter bleekeri*	+	+	
22. *Pseudohemiculter dispar*	+	+	
23. *Chanodichthys erythropterus*	+	+	
24. *Chanodichthys dabryi*		+	+
25. *Sinibrama macrops*	+	+	+
26. *Pseudolaubuca sinensis*	+		
27. *Erythroculter dabryi*		+	
28. *Erythroculter ilishaeformis*	+	+	+
29. *Rasborinus lineatus*	+		
30. *Opsariichthys bidens*		+	+
31. *pseudorasbora parva*	+	+	+
32. *Hemibarbus maculatus*	+	+	+
33. *Hemibarbus labeo*	+	+	+
34. *Pseudogobio vaillantii*	+	+	+
35. *Squalidus argentatus*		+	+
36. *Gnathopogon argentatus*	+	+	+
37. *Gnathopogon wolterstorffi*	+		
38. *Rhinogobio typus*	+		
39. *Saurogobio dabryi*		+	+
40. *Cirrhinus molitorella*	+	+	+
41. *Cirrhinus mrigala**		+	
42. *Osteochilus salsburyi*	+	+	+
43. *Distoechodon compressus*	+	+	+
44. *Xenocypris davidi*	+	+	+

45. *plagiognathops microlepis*	+	+	
46. *Aristichthys nobilis*	+	+	+
47. *Hypophthalmichthys molitrix*	+	+	+
48. *Misgurnus anguillicaudatus*		+	+
49. *Misgurnus mizolepis*		+	
50. *Copaeto semifasciolata*	+	+	+
51. *Spinibarbus hollandi*	+		
52. *Varichorhinus lepturus*			+
53. *Rhodeus ocellatus*	+	+	+
54. *Acanthorhodeus taenianalis*	+	+	
55. *Zacco platypus*	+	+	+
56. *Zacco macrolepis*	+	+	
57. *Cobitis taenia*		+	
58. *Pseudogastromyzon fasciatus jiulongjiangensis*			+
59. *Barbatula fasciolata*			+
Ⅱ **Siluriformes**			
60. *Hemibagrus macropterus*	+	+	
61. *Pterygoplichthys pardalis**	+	+	+
62. *Clarias fuscus*	+	+	
63. *Clarias batrachus**	+	+	+
64. *Silurus asotus*		+	+
65. *Leiocassis crassilabris*	+	+	
66. *Leiocassis tenuis*		+	
67. *Leiocassis albomarginatus*			+
68. *Leiocassis tenuifurcatus*			+
69. *Pelteobagrus fulvidraco*	+	+	+
70. *Pelteobaggrus nitidus*	+	+	+
71. *Pelteobagrus vachelli*	+	+	
72. *Arius sinensis*	+	+	+
Ⅲ **perciformes**			
73. *Lateolabrax japonicus*		+	+
74. *Anabas testudineus*		+	
75. *Channa argus*	+	+	
76. *Channa asiatica*			+
77. *Siniperca scherzeri*	+		
78. *Oreochromis niloticus**	+	+	+
79. *Coptodon zillii**	+	+	+
80. *Sarotherodon galilaeus**	+	+	+
81. *Oreochromis aureus**	+	+	+
82. *Oreochromis mossambicus**	+	+	+
83. *Parachromis managuensis**		+	+
84. *Rhinogobius* giurinus	+	+	+
85. *Glossogobiuss giuris*	+	+	+
86. *Odontamblyopusrubicundus*		+	
87. *Acentrogobius hoepplii*	+		
88. *Triaenopogon barbaius*		+	+
89. *Chaeturichthys stigmatias*		+	+
90. *Trypauchen vagina*	+		

91. *Eleotris oxycephala*		+	+
92. *Prionbutis koilomatodon*			
93. *Terapon jarbua*		+	
94. *Macropterus salmoides**			+
**IIII Clupeiformes**			
95. *Sardinella fimbriata*		+	
96. *Clupanodon thrissa*	+	+	+
97. *Clupanodon punctatus*	+	+	+
98. *Coilia grayi*	+	+	+
99. *Coilia mystus*		+	
**V Mugiliformes**			
100. *Liza carinata*	+	+	
101. *Mugil cephalu*	+		
102. *Mugil ophuyseni*	+	+	+
**ⅤI Tetraodontiformes**			
103. *Takifugu ocellatus*	+	+	
**ⅥI Anguilliformes**			
104. *Pisoodonophis cancrivorous*		+	
**Ⅷ Elopiformes**			
105. *Elops machnata*	+		

"+": Representing the fish species appearing in the Jiulong River Basin during each the flow seasons. "*": Representing the non-native fish species of the Jiulong River Basin.

Table S2. Fish Composition of the Jiulong River Basin.

Species	Quantity	Weight
Coptodon zillii	1311	62275.01
Sarotherodon galilaeus	248	57421.23
Oreochromis niloticus	174	17592.35
Carassius auratus	210	30682.89
Pterygoplichthys pardalis	152	33539.22
Distoechodon compressus	299	19605.3
Hemiculter leucisculus	311	11002.17
Hypophthalmichthys molitrix	83	22253.4
Pseudogobio vaillantii	202	8053.95
Pelteobagrus fulvidraco	81	9291.91
Acrossocheilus wenchowensis	202	5943.62
Rhinogobius giurinus	149	411.53
Oreochromis aureus	101	4629.29
Aristichthys nobilis	54	11501.37
Erythroculter ilishaeformis	61	2656.95
Clarias batrachus	36	10410.7
Cyprinus carpio	13	8329.11
Glossogobiuss giuris	125	1857.74
Parachromis managuensis	45	2565.6
Zacco platypus	82	1050.31
Xenocypris davidi	38	4361.03
Opsariichthys bidens	38	1442.23

Gnathopogon argentatus	45	395.17
Squaliobarbus curriculus	46	4576.2
Coilia grayi	65	1945.1
Arius sinensis	115	6498.14
Clupanodon punctatus	56	5903.36
Pelteobaggrus nitidus	30	832.49
Cirrhinus molitorella	7	4161.4
Coilia mystus	83	2505.6
Erythroculter dabryi	13	1260.6
Ctenopharyngodon idellus	13	2484.69
Chanodichthys erythropterus	24	772.8
Hemibarbus maculatus	12	1356.57
Hemibarbus labeo	15	820.96
Sarcocheilichthys nigripinnis	10	517.44
Rhodeus ocellatus	28	100.7
Plagiognathops microlepis	9	2004.25
Hemiculter bleekeri	30	674.19
Leiocassis albomarginatus	13	315.4
Clupanodon thrissa	11	866.8
Pseudohemiculter dispar	12	741.97
Squalidus argentatus	20	103
Odontamblyopusrubicundus	27	320.5
Saurogobio dabryi	9	333.2
Copaeto semifasciolata	15	48.6
Eleotris oxycephala	12	224.8
Acrossocheilus fasciatus	10	265.38
Chanodichthys dabryi	10	242.4
Takifugu ocellatus	10	603.2
Misgurnus anguillicaudatus	7	127.31
Acrossocheilus fasciatus	6	288.35
Pseudorasbora parva	9	52.5
Clarias fuscus	6	384.95
Leiocassis crassilabris	2	616.3
Mugil cephalu	2	1264.9
Liza carinata	4	261.1
Anabas testudineus	5	173.2
Sinibrama macrops	6	84.54
Varichorhinus lepturus	6	13.5
Silurus asotus	2	317.8
Rhinogobio typus	4	165.29
Misgurnus mizolepis	5	84.5
Acrossocheilus hemispinus	7	349.3
Sarcocheilichthys kiangsiensis	8	209.6
Channa argus	4	464.65
Osteochilus salsburyi	6	227.9
Hemibagrus macropterus	3	106.67

Acanthorhodeus taenianalis	4	19.8
Pelteobagrus vachelli	2	169.1
Mugil ophuyseni	2	151.8
Channa asiatica	3	58.4
Chaeturichthys stigmatias	6	110.6
Acentrogobius hoepplii	7	14
Triaenopogon barbaius	6	54.4
Lateolabrax japonicus	3	280.9
Rhodeus sinensis	3	6.2
Macropterus salmoides	2	68.8
Prionbutis koilomatodon	5	46.9
Onychostoma barbatulum	4	121.24
Rasborinus lineatus	2	45.37
Oreochromis mossambicus	2	23.15
Leiocassis tenuifurcatus	1	257.5
Cobitis taenia	2	12.3
Spinibarbus hollandi	1	192
Sardinella fimbriata	2	28.6
Pseudobrama simoni	1	99.6
Sarcocheilichthys sinensis	1	87.8
Cirrhinus mrigala	1	84.5
Siniperca scherzeri	1	78.58
Mylopharyngodon piceus	1	66.2
Elops machnata	1	57.9
Sarcocheilichthys sinensis fukiensis	1	52.17
Pisoodonophis cancrivorous	1	39.9
Acrossocheilus kreyenbergii	1	28.4
Pseudolaubuca sinensis	1	27.2
Leiocassis tenuis	1	20.2
Zacco macrolepis	1	16.6
Terapon jarbua	1	11.3
Trypauchen vagina	1	9
Aphyocypris chinensis	1	4.6
Pseudogastromyzon fasciatus jiulongjiangensis	1	4.5
Abbottina rivularis	1	3.55
Barbatula fasciolata	1	2.9
Gnathopogon wolterstorffi	1	2.8
Total	4933	375302.94

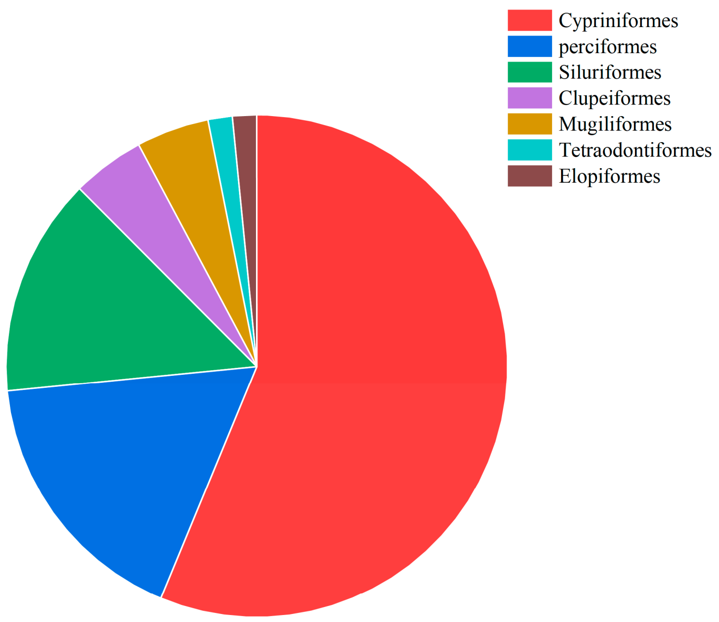

Figure S1. The percentage of order richness during the low flow season in the Jiulong River Basin.

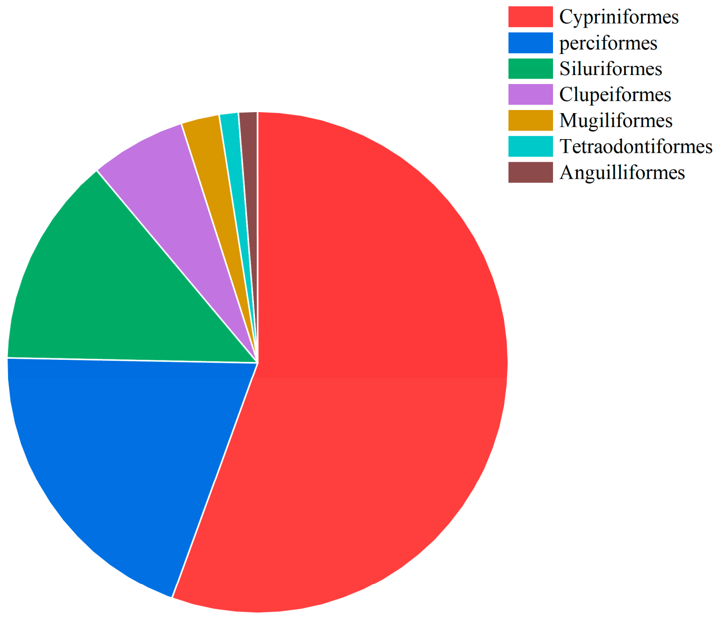

Figure S2. The percentage of order richness during the normal flow season in the Jiulong River Basin.

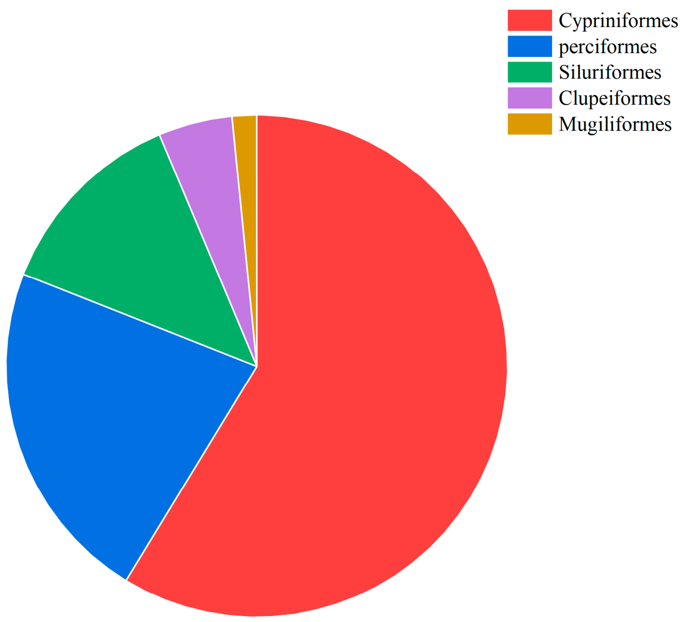

Figure S3. The percentage of order richness during the high flow season in the Jiulong River Basin.

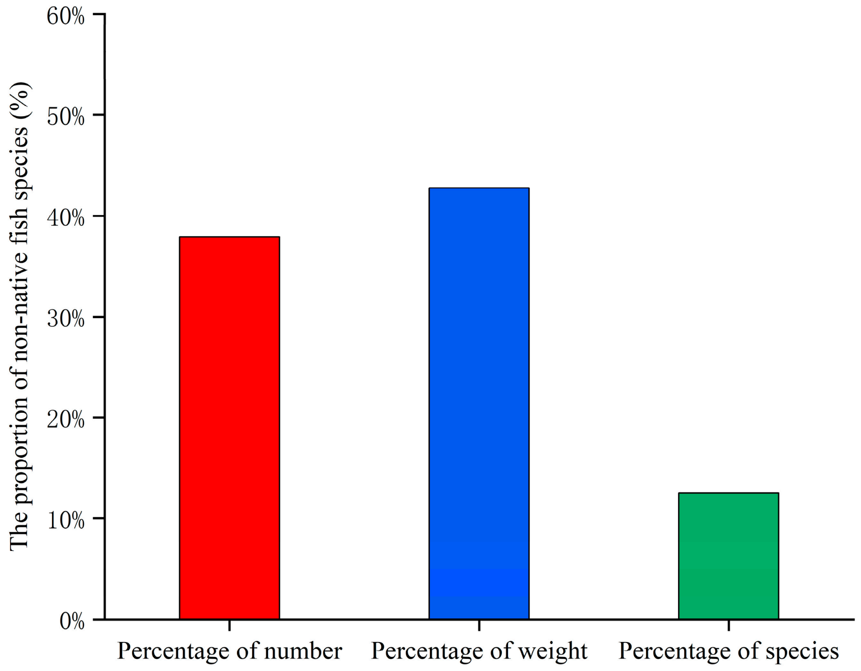

Figure S4. The individual number, weight and species richness Percentage of non-native fish during the low flow season in the Jiulong River Basin.

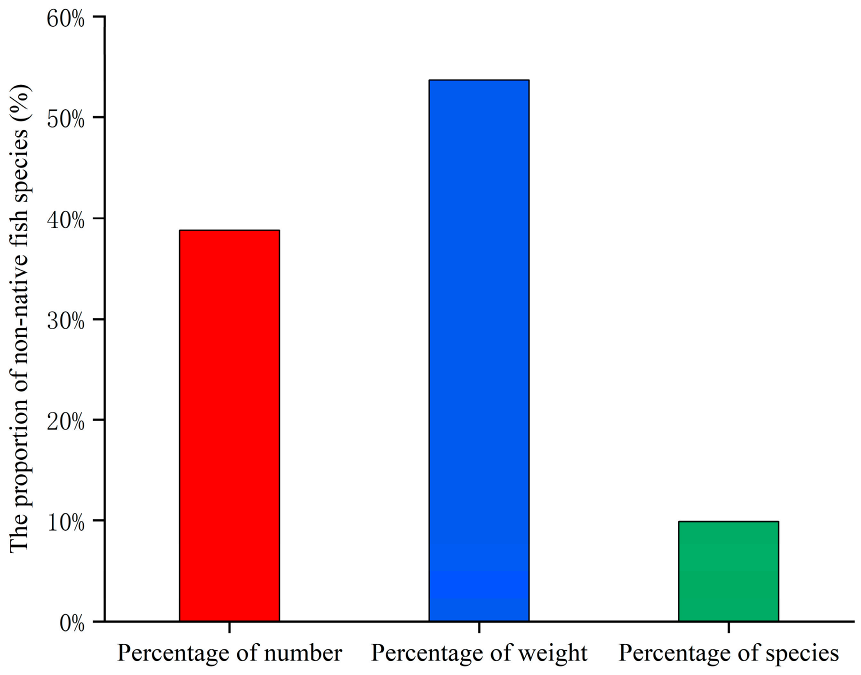

Figure S5. The individual number, weight and species richness Percentage of non-native fish during the normal flow season in the Jiulong River Basin.

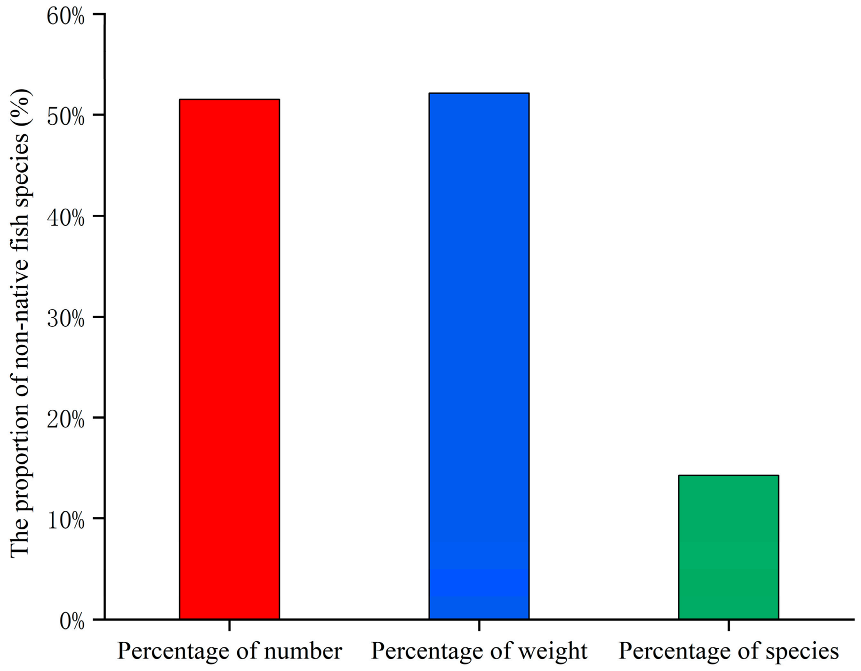

Figure S6. The individual number, weight and species richness Percentage of non-native fish during the high flow season in the Jiulong River Basin.
